# The Physicochemical Characterization of New “Green” Epoxy-Resin Hardener Made from PET Waste

**DOI:** 10.3390/polym14204456

**Published:** 2022-10-21

**Authors:** Grigorii K. Sterligov, Sergey A. Rzhevskiy, Dilshodakhon K. Isaeva, Nikita M. Belov, Maria A. Rasskazova, Egor A. Drokin, Maxim A. Topchiy, Lidiya I. Minaeva, Alexander V. Babkin, Erdni M. Erdni-Goryaev, Alexey V. Kepman, Andrey F. Asachenko

**Affiliations:** 1 A. V. Topchiev Institute of Petrochemical Synthesis, Russian Academy of Sciences, 119991 Moscow, Russia; 2 Faculty of Chemistry, National Research University Higher School of Economics, 101100 Moscow, Russia; 3 Department of Chemistry, M. V. Lomonosov Moscow State University, 11991 Moscow, Russia

**Keywords:** polyethylene terephthalate, PET waste, epoxy matrix, new hardener, recycling

## Abstract

“Green” thermally stable hardener was synthesized from a PET waste. The rigid molecular linear structure of the new hardener suggests that it will provide the polymer matrix with the necessary physical and mechanical characteristics. It also allows the expectation that cured matrix based on this hardener can provide increased toughness. New hardener was used as a curing agent for three epoxy resins—tetraglycidyl methylenedianiline (TGDMA, 111–117 EEW), diglycidylether of bisphenol A (DGEBA, 170-192 EEW) and solid epoxy resin (SER)—with a medium molecular weight (860–930 EEW) based on DGEBA. The mixtures were found to have the highest T_g_ for the DGEBA resin, and high of that for TGDMA and SER. According to the DMA analysis for two cured matrices, the hardener proved to be no worse than the standard ones, and made it possible to obtain cured matrices with excellent mechanical properties, which allows us to hope for further application of new hardener cured epoxy matrices in appropriate composite materials at high temperatures.

## 1. Introduction

Epoxy resins are most widely used for resin based composites in the aerospace/aircraft industry [1,2,3] where high service temperature is required. These resins should possess high thermal stability, convenient processability and good mechanical properties, along with chemical resistance. In this case, new hardeners [4,5,6,7] for polyepoxides for composite materials are of particular interest because the hardener structure affects the final properties of the cured polymer matrix in equal measure with the epoxy resin.

The main parameter of interest for epoxy resin hardeners is a high glass transition temperature (T_g_) of the cured polymer matrix, as it allows to use materials at elevated temperatures. Currently, a fairly limited number of hardeners are used to ensure a high T_g_, in particular, mainly represented by 4,4′-diaminodiphenyl sulfone (DDS) [8,9,10,11] and dicyandiamide (DICY) [12,13,14,15]. The reason for this selectivity is the stringent requirements for final mixture of resins, hardeners and other functional additives (tougheners, pigments, rheological additives, etc.), in particular the mixture should be able to be stored for a long time without curing and to cure with sufficient speed at a certain temperature. The disadvantages of DDS and DICY include the fragility of the polymer matrices obtained with them, which is unacceptable for aviation, as well as the need to use special accelerator additives [16,17]. For mass application, price and synthetic availability are also very important aspects. Therefore, the development of new hardener for epoxy matrices with desired properties and high T_g_ with inherent toughness made from cheap and accessible materials is a very urgent task.

The widespread daily consumption of PET-made (polyethylene terephthalate) products in modern life has led to an acute problem of PET waste disposal [18] because PET is known to be non-biodegradable in nature. That is why the recycling of PET [19] has become one of the most important scientific topics regarding that only 28.4% of PET is recycled [20]. The search for new value-added and useful products from PET waste led to some attempts to make the epoxy resins [21,22], and new hardeners [23]. These preconditions led us to the idea of obtaining a new hardener with the desired properties from PET.

Herein we report a new hardener for epoxy matrices, obtained directly from PET products, for which its properties and interaction with commonly encountered epoxy resins have been studied.

## 2. Materials and Methods

### 2.1. Materials

Epoxy resins used in this study were commercially available industrial standard products. Tetraglycidyl methylenedianiline (TGDMA) with epoxy equivalent weight (EEW) 111–117 was purchased from Huntsman Advanced Materials, Monthey, Switzerland as Araldite MY 721 and was use as received without further purification, diglycidyl ether of bisphenol A (185–192 EEW) (DGEBA) was purchased from Hexion Inc., Rotterdam, The Netherlands as EPON Resin 828 and solid epoxy resin (SER) with medium molecular weight (860–930 EEW) based on DGEBA was purchased from Olin Corp. Clayton, NC, USA as D.E.R. 664UE and was use as received. m-Phenylenediamine, 1,5,7-triazabicyclo [4.4.0]dec-5-ene, THF, methylene chloride and methanol were obtained from Aldrich. PET waste was collected from post-consumer bottles.

#### 2.1.1. N^1^,N^4^-bis(3-aminophenyl)terephthalamide (MPD-TPA-MPD) Hardener Synthesis from PET Waste

The synthesis of *N^1^,N^4^*-bis(3-aminophenyl)terephthalamide (MPD-TPA-MPD) was performed according to known procedure [24]. PET bottles were crushed to small flakes after washing with soap and methanol. Well ground PET flakes (5.3 g, 27.6 mmol), *m*-phenylenediamine (48.4 g, 0.45 mol) and 1,5,7-triazabicyclo[4.4.0]dec-5-ene (200 mg, 1.4 mmol) were heated with stirring under argon atmosphere in Schlenk flask at 190 °C for 18 h. The homogeneous solution was then poured in 1000 mL of THF and filtered. The filtrate was concentrated and precipitated in water with stirring. The residue was washed with methylene chloride a few times and dried in vacuo, yielding a pale pink powder as *N*^1^,*N*^4^-bis(3-aminophenyl)terephthalamide MPD-TPA-MPD (6.87 g, 72%).

^1^H NMR (400 MHz, DMSO-*d6*): δ 10.07 (s, 2H, NH), 8.04 (s, 4H, Ar-H), 7.12 (s, 2H, Ar-H), 6.98 (t, *J* = 7.9 Hz, 2H, Ar-H), 6.89 (d, *J* = 7.4 Hz, 2H, Ar-H), 6.34 (d, *J* = 7.7 Hz, 2H, Ar-H), 5.10 (s, 4H, NH).

^13^C [22] NMR (101 MHz, DMSO) δ 164.6, 149.0, 139.6, 137.6, 128.8, 127.6, 110.0, 108.4, 106.2. The NMR data are in good correspondence with the literature.

#### 2.1.2. N^1^,N^4^-bis(3-aminophenyl)terephthalamide (MPD-TPA-MPD) Hardener Synthesis from PET Waste Using Methanol and Water

The synthesis was performed according to procedure 2.2.1. After stirring under argon atmosphere at 190 °C for 18 h the reaction mixture was cooled to ca. 70 °C, poured in 500 mL of methanol and filtered. The solid residue was discarded, the filtrate was concentrated in vacuo to 50 mL, diluted with hot water (400 mL) and filtered. The residue was washed 3 times with 100 mL of hot water followed by hot methanol (150 mL) and dried in vacuo, yielding a pale pink powder as *N*^1^,*N*^4^-bis(3-aminophenyl)terephthalamide MPD-TPA-MPD (4.31 g, 45%). Concentration of methanol filtrate and repeated treatment using reduced volumes of solvents allowed to obtain additional portion of MPD-TPA-MPD (1.82 g, 19%).

### 2.2. Epoxy Matrices Preparation

The epoxy matrices were prepared using commercially available epoxy resins TGDMA, DGEBA, and SER mixed with *N*^1^,*N*^4^-bis(3-aminophenyl)terephthalamide (MPD-TPA-MPD) in a following mass ratios: TGDMA: MPD-TPA-MPD—5:3.89; DGEBA: MPD-TPA-MPD—5:2.54; SER: MPD-TPA-MPD—5:0.481. The mass ratios were based on EEW and AHEW, MPD-TPA-MPD was considered as a diamine and amine equivalent weight (AHEW) was accepted as 86.5 g/eq. Regarding the resins, the EEW were calculated as the following: EEW (TGDMA)—111 g/eq, EEW (DGEBA)—170 g/eq, and EEW (SER)—900 g/eq. For differential scanning calorimetry (DSC) and simultaneous thermal analysis (STA), analysis resins were thoroughly mixed with MPD-TPA-MPD without dissolution. Regarding solid SER the resin was ground with diamine to a homogeneous state.

To determine the storage modulus of and glass transition temperature by the dynamic mechanical analysis (DMA) method, samples of cured resins were made in special rectangular mold. The hardener dissolves only above 130 °C; the dissolution is accompanied by rapid gelation. Therefore, for the production of samples, the resin was thoroughly mixed with diamine without dissolution. In addition, in the sample with SER, the solid resin was ground with diamine to a homogeneous state. This mixture was placed on one of the plates with a frame and preheated in an oven to 110 °C to distribute viscous or semi solid mixture on the stainless-steel mold coated by release agent with cavity for the resin plate 100 × 100 × 2 mm^3^ surface (length × width × thickness). Then, the mold with the resin was placed in a desiccator and kept under vacuum for 15 min to remove dissolved air and residual moisture from the resin to obtain specimen without defects such as air bubbles. After that, the first plate was covered with the second plate, and fasteners were installed around the perimeter to obtain final defined thickness. Curing was provided with following schedule: (1) heating to 110 °C with rate 2 °C/min; (2) hold 110 °C 0.5 h; (3) heating to 180 °C with rate 2 °C/min; (4) hold 180 °C 3 h; and (5) cooling to room temperature with rate 2 °C. Curing was provided in an air-circulated, heated oven. The resulting cured plates were cut by CNC machine to obtain rectangular specimens with dimensions of 100 × 100 × 2 mm^3^ (length × width × thickness).

### 2.3. Methods

Differential scanning calorimetry (DSC) experiments were carried out on a DSC TA Instruments Q20 Differential Scanning Calorimeter (TA Instruments, New Castle, DE, USA) completed with controlled by Nautilus personal computer with “Advantage” software (version 5.5.24, TA Instruments New Castle, DE, USA). Compounds or freshly prepared mixtures of about 5–7 mg were placed into aluminum crucibles in an inert nitrogen atmosphere with a purity of 99.6% with a flow rate of 100 mL/min in a temperature range from 50 to 325 °C, followed by cooling to 50 °C, with a heating rate of 10 °C/min. The device was calibrated for indium and tin.

Simultaneous thermal analysis (STA) experiments were carried out on a STA 449 F5-Jupiter apparatus (NETZSCH, Selb, Germany) completed with controlled by personal computer with Netzsch Proteus software (version 6.0.1, NETZSCH, Selb, Germany). The measurements were carried out in aluminum crucibles in a nitrogen atmosphere with a flow rate of 70 mL/min in the temperature range from 30 to 600 °C, with a heating rate of 10 °C/min. A S type sensor was used for TG/DSC measurements. The instrument is calibrated for indium, tin and zinc.

Dynamic mechanical analysis (DMA) was carried out on a DMA Q800 analyzer (TA Instruments, New Castle, DE, U.S.), controlled by personal computer Nautilus with software “Advantage” (version 5.5.24, TA Instruments New Castle, DE, USA). The measurements were carried out in air in the temperature range from 50 to 400 °C, with a heating rate of 5 °C/min with 3-Point Bending method. The specified amplitude is 20 µm, the frequency is 1 Hz.

NMR spectra were obtained on a Bruker “Ascend 400” (400 MHz ^1^H, 101 MHz ^13^C) Bruker (Billerica, Massachusetts, U.S.). The chemical shifts were frequency referenced relative to the residual undeuterated solvent peaks. Coupling constants *J* are given in Hertz as positive values regardless of their real individual signs. The multiplicity of the signals is indicated as ‘‘s”, ‘‘d”, “t” or ‘‘m” for singlet, doublet, triplet or multiplet, respectively.

## 3. Results

### 3.1. MPD-TPA-MPD Hardener Synthesis from PET Waste

**Figure 1:**
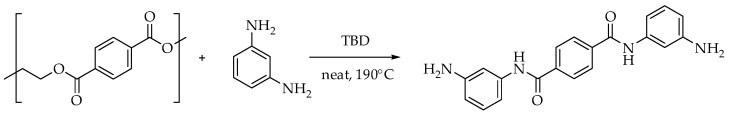
MPD-TPA-MPD synthesis from PET waste.

To synthesize the new “green” hardener from PET waste it was decided to use the known procedure [24] of the PET waste aminolysis, using PET flakes, *m*-phenylenediamine excess and 1,5,7-triazabicyclo[4.4.0]dec-5-ene (TBD) as a catalyst (Figure 1). Aminolysis product *N*^1^,*N*^4^-bis(3-aminophenyl)terephthalamide (MPD-TPA-MPD) was obtained in 69% yield.

### 3.2. Differential Scanning Calorimetry (DSC) of MPD-TPA-MPD

**Figure 2:**
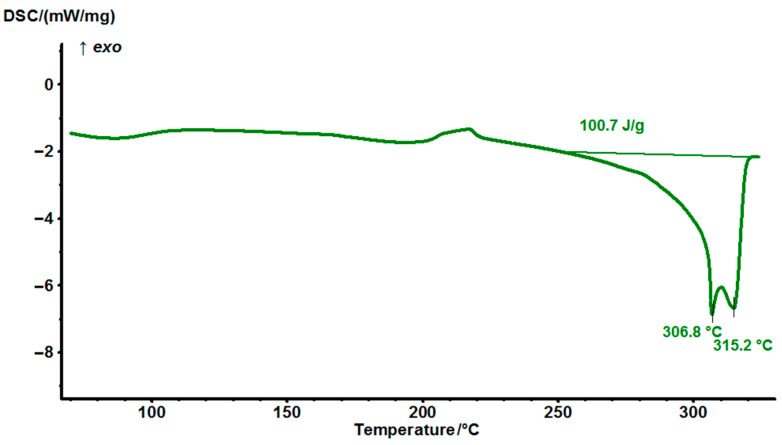
DSC thermograms of MPD−TPA−MPD at a heating rate of 10 °C/min.

To determine the melting temperature of the obtained MPD-TPA-MPD “green” hardener, a DSC analysis was carried out in a nitrogen atmosphere in the heating mode (heating rate of 10 °C/min) in the temperature range of 50–350 °C. The results of the study are presented in Figure 2.

The melting temperature of the hardener was determined as 315 °C by means of DSC.

### 3.3. Simultaneous Thermal Analysis (STA) of MPD-TPA-MPD in a Nitrogen Atmosphere and an Air Atmosphere

**Figure 3:**
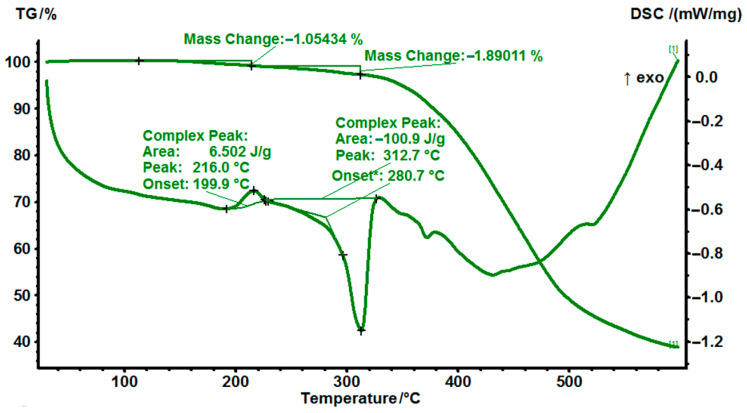
STA−thermograms of MPD−TPA−MPD in nitrogen atmosphere at a heating rate of 10 °C/min.

**Figure 4:**
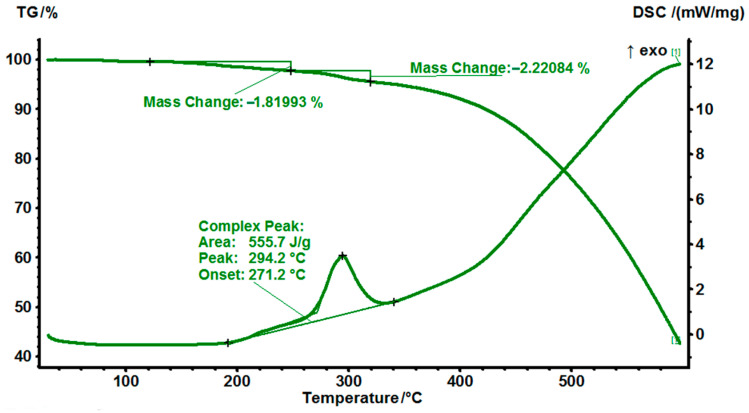
STA−thermograms of MPD−TPA−MPD in the air atmosphere at a heating rate of 10 °C/min.

Moreover, to determine the initial stability of the compound, a comparison of simultaneous thermal analysis (STA) results for MPD-TPA-MP in a nitrogen atmosphere (Figure 3) and an air atmosphere (Figure 4) was made. The samples were dried under vacuum at room temperature before analysis.

As can be seen from the STA curve in Figure 3, the MPD-TPA-MPD “green” hardener is a crystalline structure with a melting point of 315 °C that is in good correspondence with the DSC analysis. The compound also partially contains an amorphous phase, which begins to crystallize at an elevated temperature causing a small exothermic peak before the onset of melting. The noticeable rate of decomposition of the hardener begins above 350 °C, which makes it suitable for the high-temperatures technological application.

The STA-thermograms of MPD-TPA-MPD in the air atmosphere turned out to be significantly different from ones in the nitrogen atmosphere (Figure 4). In the air atmosphere, there is a large exo-peak having a small mass loss, which was not observed in the nitrogen atmosphere. The peak temperature approximately corresponds to the hydrogen bond breaking temperature (about 300 °C). Apparently, in an inert atmosphere, the breaking of hydrogen bonds in the sample occurs synchronously with its melting. While in the air atmosphere, after the rupture of hydrogen bonds, the oxidation process begins to proceed without significant loss of mass. An explanation for this phenomenon can be both the formation of intermolecular nitrogen–nitrogen bonds with oxidation of two hydrogen atoms or possible intramolecular cyclization with the formation of a bis-oxazole structure, which occurs with a large release of energy due to aromatization

After a complete characterization of the “green” hardener MPD-TPA-MPD, it became clear that the substance obtained from PET waste is stable, partially amorphous, has a high melting point, suitable for epoxy matrix curing. It is shown that the thermal stability of MPD-TPA-MPD hardener makes it possible to use it for epoxy resins with high curing temperature. In case of low molecular weight of the epoxy resins, the structure of the hardener should have a significant impact on the cured matrix properties and its rigid molecular linear structure suggests that it will provide the polymer matrix with the necessary physical and mechanical characteristics, rigidity (reduce brittleness and increase the strength of the polymer matrix) and high glass transition temperature in the final polymer matrix.

### 3.4. DSC Analysis of Different Epoxy Resins Compositions with MPD-TPA-MPD as a Hardener

Regarding good MPD-TPA-MPD thermal analysis results, three typical formulations with different epoxy resins were obtained for further investigations. The structural formulas, the resins names and the hardener/resin mass ratio are presented in Table 1. TGDMA was chosen due to the fact that it provides the one of the highest glass transition temperatures of the matrix and DGEBA is the most commonly used epoxy resin. It was also interesting to investigate the behavior of “green” hardener in the composition with SER, because this epoxy resin is usually evaluated in the powder coatings production. In this field shelf life of the resin and hardener composition becomes an important criterion, so the search for so-called “latent” (slow working) hardener is a very relevant task. Factors such as increased elasticity of the paint along with the “green” nature of the hardener, made from plastic waste, can also be pointed out as critical for the new hardeners search. It should be noted that TGDMA and DGEBA resins are viscous liquids at room temperature, while SER resin is solid flakes. These properties determine a slight difference in the preparation of compositions with them.

To determine curing behavior the mixtures of hardener MPD-TPA-MPD with different epoxy resins DSC analysis was carried out in a nitrogen atmosphere in the heating mode (heating rate of 10 °C/min) in the temperature range of 50–280 °C. Liquid resins TGDMA and DGEBA were mixed with hardener with heating. Initially, MPD-TPA-MPD powder was tried to be dissolved in resins. The diamine began to dissolve only at 130 °C, but almost immediately the components began to react and the mixtures gelled. Therefore, for DSC analysis, the resins were thoroughly mixed with diamine without dissolution. In addition, regarding the EG531 composition the solid resin was ground with diamine to a homogeneous state.

**Figure 5:**
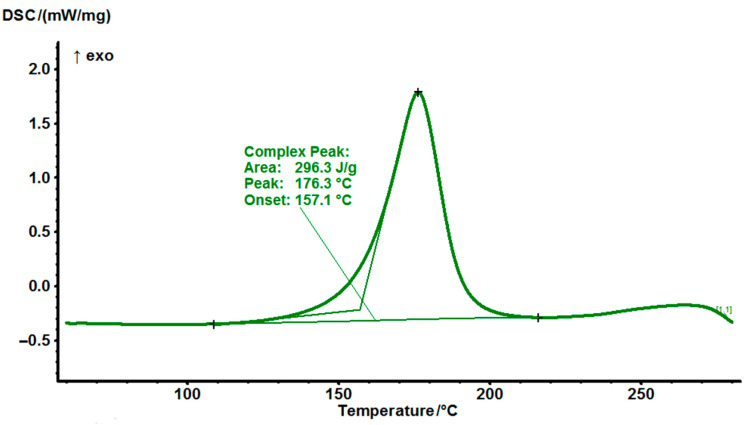
DSC thermograms of non−cured EG513 at a heating rate of 10 °C/min.

The results of the study for EG513 composition are presented in Figure 5. There is an exotherm peak of the EG513 composition and it is found to be 176 °C according to DSC.

All three mixtures of epoxy resins with MPD-TPA-MPD were cured at 180 °C for 3 h. In all cases, the reaction starts at a sufficiently high temperature. The total peaks areas are not large so it may be concluded that there will be no explosive curing mechanism. After the curing process was stopped the DSC analysis was also made for all 3 the cured samples.

**Figure 6:**
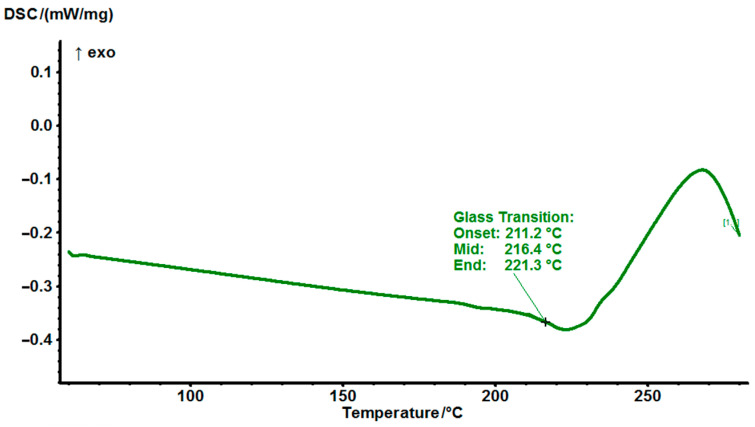
DSC thermograms cured EG513 at a heating rate of 10 °C/min.

In case of cured EG513 the post cure peak after T_g_ was found on DSC at 216 °C (Figure 6). That result indicates that the 180 °C temperature is not sufficient to cure the EG513 mixture, which has a higher T_g_ and the components don’t have opportunity to react thoroughly in a partially hardened matrix. The T_g_ was determined here as 216 °C, which can be defined as high regarding this resin type. Due to not complete curing of this mixture at the typical temperature of 180 °C, further works with this mixture nor carried out.

**Figure 7:**
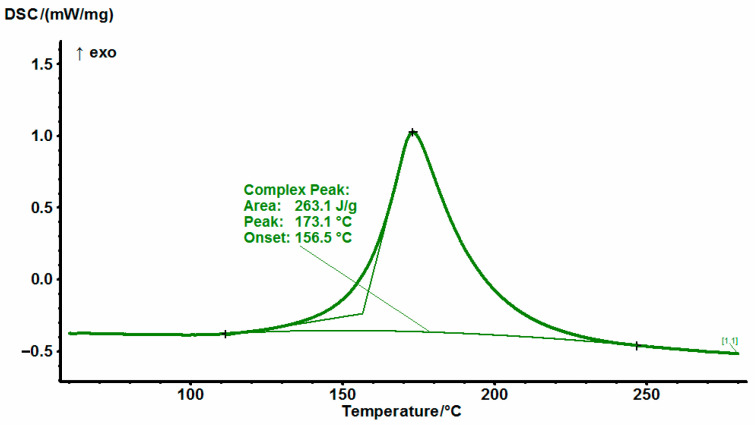
DSC thermograms of non-cured EG514 at a heating rate of 10 °C/min.

**Figure 8:**
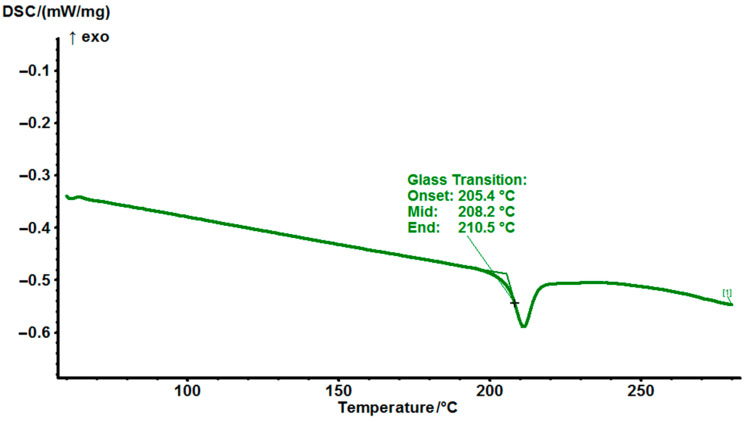
DSC thermograms cured EG514 at a heating rate of 10 °C/min.

Regarding DGEBA, the T_g_ is found to be 208 °C and the resin is fully cured at this temperature (Figure 7 and Figure 8). To the best of our knowledge, this T_g_ is the highest ever achieved for curing DGEBA with any hardener. It is much higher comparing with the commonly used DDS and DICY with T_g_ of 184 °C [25] and 138 °C [26], respectively.

**Figure 9:**
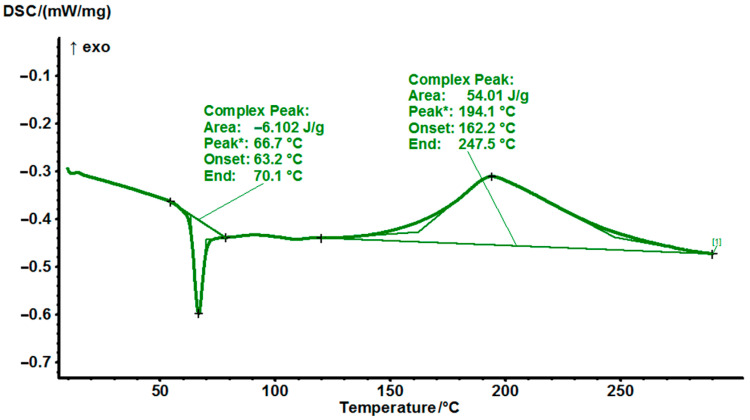
DSC thermograms of non-cured EG531 at a heating rate of 10 °C/min.

Regarding the DSC thermogram of non-cured EG531 the first peak at 67 °C corresponds to solid SER epoxy resin melting, after that the reaction occurs at 194 °C (Figure 9).

**Figure 10:**
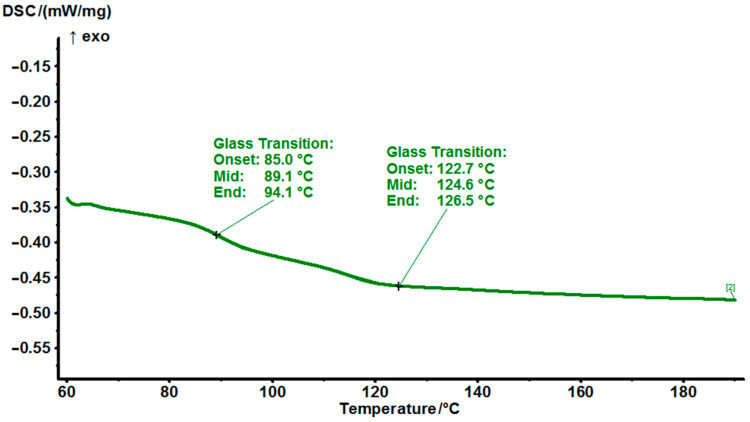
DSC thermograms cured EG531 at a heating rate of 10 °C/min.

The SER resin is fully cured using MPD-TPA-MPD as a hardener. For EG531, there are two thermal effects similar to the glass transition temperature on the DSC thermogram, and the glass transition temperature of 90 °C is characteristic for this resin with other hardeners (Figure 10).

The results of DSC analysis for all three cured with MPD-TPA-MPD epoxy resins are presented in Table 2. As a result of the MPD-TPA-MPD use as a hardener, the T_g_ for TGDMA and SER resins mixtures turned out to be quite similar with other previously studied hardener. With regard to DGEBA, the application of the new “green” hardener induced a significant increase in T_g_ compared to other commonly used compounds.

### 3.5. Dynamic Mechanical Analysis (DMA) of Cured EG514 and EG531

**Figure 11:**
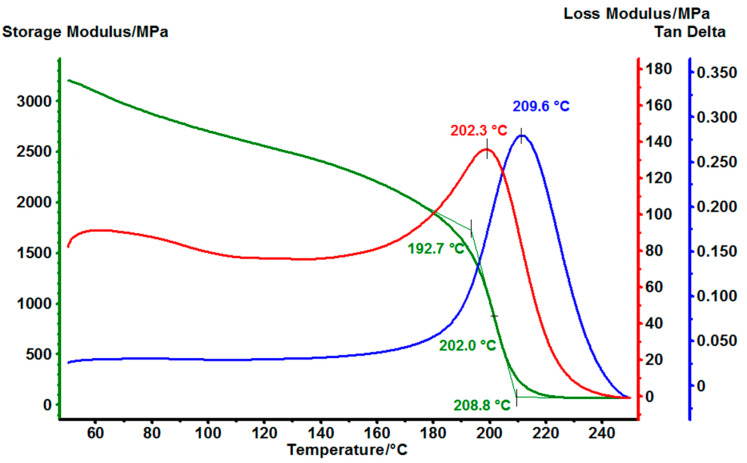
DMA thermogram of EG514.

**Figure 12:**
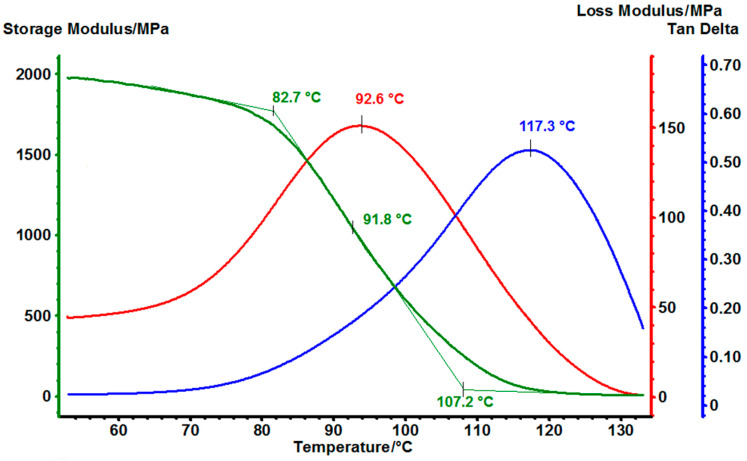
DMA thermogram of EG531.

To investigate the mechanical properties of cured epoxy matrices the dynamic mechanical analysis for samples EG514 (Figure 11) and EG531 (Figure 12) was performed. For that purpose, the samples were made especially for DMA, as a tooling consists of two plates and a frame between them. The plates were 2 mm thick. To make the EG514 sample the resin was mixed with up the hardener without dilution, and in case of EG531 the solid resin was mixed with MPD-TPA-MPD and ground in a mortar. For both samples curing was carried out according to the following regime—heating up to 110 °C at a rate of 2 °C/min, exposure at 110 °C for 30 min, heating up to 180 °C at a rate of 2 °C/min and finally exposure at 180 °C for 180 min.

For EG514 sample the correlation between storage modulus and temperature can be found out from DMA (Figure 11). At room temperature it is above 3 GPa, so the polymer matrix has excellent stiffness. With heating, the storage modulus gradually decreases up to T_g_ to 2 GPa, but still remains at a high level, and becoming very low only after T_g_. The data occur in accordance with DSC analysis. We hope that the high storage modulus will make it possible to make various composite materials based on this polymer matrix, and it will help to redistribute the load on the reinforcing fillers effectively.

The same result regarding storage modulus correlation with the temperature is observed in case of EG531 sample (Figure 12). It may be called typical for the powder coating containing SER epoxy resin, because this resin has higher molecular weight than other used in this work, and since there is relatively little hardener added, so the thermal properties of the matrix are largely determined by the properties of the resin itself [27,28]. It can be pointed out that “green” MPD-TPA-MPD hardener made from PET waste shows heat resistance and other characteristics no worse than standard DDS with the same epoxy resin [29] and does not require the use of a reaction accelerator.

The results of DMA analysis for all 2 cured with MPD-TPA-MPD epoxy resins are presented in Table 3.

## 4. Conclusions

In this work “green” MPD-TPA-MPD hardener was synthesized from a PET waste and its thermal stability and properties were investigated by means of DSC. It was shown that the hardener is thermally stable and can be used at high temperatures. Moreover, from one side rigid molecular linear structure suggests that it will provide the polymer matrix with the necessary physical and mechanical characteristics from other side linear internal structure of the hardener allow us to expect that cured matrix based on this hardener can provide increase toughness. MPD-TPA-MPD was used as a curing agent for three epoxy resins. The mixtures obtained and the cured matrices were examined by DSC and found to have the highest T_g_ for the DGEBA resin, for the other two it was at the level of standard polymer matrices with other hardeners. For two cured matrices, DMA studies were performed to investigate their physical and mechanical properties when heated. In all cases, the hardener proved to be no worse than the standard ones, and made it possible to obtain cured matrices with excellent mechanical properties. In particular, the storage modulus is quite large and decreases insignificantly up to T_g_, which allows us to hope for further application of MPD-TPA-MPD cured epoxy matrices in appropriate composite materials at high temperatures.

## Figures and Tables

**Table 1:** Epoxy resins used for compositions with MPD-TPA-MPD hardener and the hardener/resin mass ratio.

Sample Name	Epoxy Resin Name	Formula	Hardener/Resin Mass Ratio
EG513	TGDMA 4,4′-Methylenebis(N,N-diglycidylaniline)	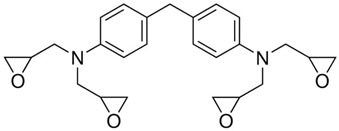	3.89/5
EG514	DGEBA Bisphenol A diglycidyl ether	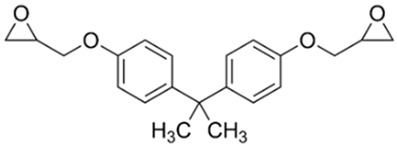	2.54/5
EG531	SER Poly(Bisphenol A-co-epichlorohydrin), glycidyl end-capped, n ≈ 4	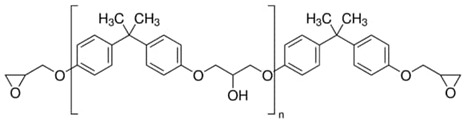	0.481/5

**Table 2:** T_g_ of cured samples due to the DSC analysis.

Sample	T_g_, °C
EG513	216
EG514	208
EG531	89 (124)

**Table 3:** T_g_ of cured samples due to the DMA analysis.

Sample	Tg, °C
EG514	202
EG531	91.8
